# Hyperoside Modulates the Estrogen‐PI3K/VEGF Axis to Ameliorate Oxidative Damage‐Induced Inhibition of Bone Formation in MC3T3‐E1 Cells

**DOI:** 10.1002/iid3.70412

**Published:** 2026-04-06

**Authors:** Shuo Wang, Wei Feng, Xueqin Feng, Peitong Wu, Nanxi Zhang, Xiaoqian Yang, Yawen Li, Chunnan Li, Jiaming Sun

**Affiliations:** ^1^ Jilin Ginseng Academy Changchun University of Chinese Medicine Changchun China; ^2^ School of Pharmaceutical Sciences, Quality Evaluation & Standardization Hebei Province Engineering Research Center of Traditional Chinese Medicine Hebei University of Chinese Medicine Shijiazhuang China

**Keywords:** hyperoside, MC3T3‐E1 cells, osteoporosis, oxidative stress, zebrafish

## Abstract

**Objective:**

This study aims to investigate how hyperoside (HYP) alleviates oxidative stress‐induced osteoporosis, its molecular mechanisms, and its impact on osteoblast differentiation, oxidative damage, and the estrogen‐PI3K/VEGF signaling pathway.

**Methods:**

The osteoblast differentiation model was induced using dexamethasone, and osteoblast‐related markers like ALP, NO, GSH, MDA, and SOD were measured post‐HYP intervention. A zebrafish model was used to assess HYP's impact on ROS and bone formation. Network pharmacology identified key oxidative stress and osteoporosis targets, with HYP's binding affinity confirmed via molecular docking and simulation. RT‐qPCR verified the expression of key pathway targets.

**Results:**

HYP can counteract dexamethasone‐induced inhibition of osteoblast differentiation, boost ALP, NO, GSH, and SOD levels, and lower MDA levels in osteoblasts. It also reduces ROS accumulation and enhances bone formation in zebrafish. Network pharmacology identified a common oxidative stress and osteoporosis target, with molecular docking confirming HYP's stable binding. RT‐qPCR showed HYP significantly upregulates SRC, PI3K, AKT1, and e‐NOS, activating the estrogen‐PI3K/VEGF pathway.

**Conclusion:**

HYP plays an anti‐oxidative stress effect through targeted regulation of estrogen‐PI3K/VEGF signal axis, and then promotes osteoblast differentiation and bone formation, which provides a new potential candidate drug and experimental basis for the treatment of osteoporosis.

## Graphical abstract

This study explores the potential of hyperoside to activate the estrogen‐PI3K/VEGF axis, thereby suppressing oxidative stress and enhancing osteogenic differentiation to mitigate osteoporosis. The initial proposal of the estrogen‐PI3K/VEGF signaling pathway axis as a potential therapeutic approach for osteoporosis. The pharmacological effects of oxidative stress and osteoporosis have been confirmed through dual experiments conducted on MC3T3‐E1 cells and zebrafish.

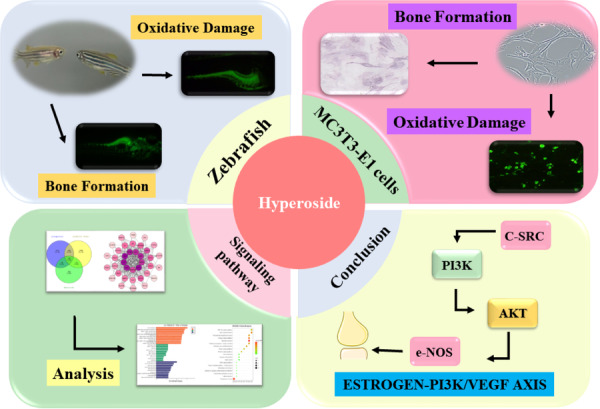

## 1 Introduction

Osteoporosis (OP) is a prevalent metabolic bone disease characterized by decreased bone mass, compromised bone tissue microarchitecture leading to increased fragility, and elevated fracture susceptibility [1]. It is recognized as a systemic condition with global prevalence. Bone metabolism, essential for the maintenance of osteoblasts and osteoclasts, is a lifelong process that ensures skeletal homeostasis [2, 3]. Osteoblasts, originating from mesenchymal stem cells, are capable of secreting a diverse array of bioactive substances. These cells are pivotal in the processes of bone formation, repair, and metabolism, and hold potential for therapeutic applications in bone diseases [4]. Disruption of the equilibrium between osteoblasts and osteoclasts may ultimately culminate in the pathogenesis of OP [5, 6]. Among these factors, oxidative stress is widely recognized as a significant contributor to the development of bone homeostasis disorders. Initially conceptualized within the framework of free radical senescence, oxidative stress has since been characterized as a pathological state characterized by an imbalance between reactive oxygen species (ROS) and the endogenous antioxidant defense system. This imbalance can result in cellular dysfunction, protein and DNA damage, and ultimately irreversible cellular harm and death [7]. In the pathophysiology of OP, dysregulated generation of ROS results in heightened oxidative stress and subsequent apoptosis of osteoblasts, prompting the differentiation of osteoclasts and disrupting the equilibrium between bone resorption and formation following osteoblast injury [8, 9]. Therefore, the search for natural antioxidant chemicals with no side effects to reduce oxidative damage and promote osteoblast differentiation has become an important goal in the treatment of OP.

Hyperoside (HYP) is a flavonoid compound with natural antioxidant properties, primarily found in hawthorn, Cuscuta and various food or medicinal plants. It exhibits antioxidant, anti‐inflammatory, and cardiovascular protective effects, and has the ability to modulate the nervous, immune, digestive, and circulatory systems [10]. Emerging research indicates that HYP possesses notable pharmacological potential as an antioxidant. For instance, HYP has been shown to protect cardiomyocytes from oxidative stress by activating the PI3K/AKT/Nrf2 signaling pathway [11, 12], and to inhibit oxidative stress induced by 6‐hydroxydopamine while activating the Nrf2/HO‐1 signaling pathway in dopaminergic neurons [13]. The antioxidant properties of HYP are integral to its mechanism of action, providing significant benefits in ameliorating oxidative stress. In their study on the treatment of OP, Yiqing Chen et al. [14] discovered that HYP effectively addressed bone metabolic disorders in ovariectomised mice and hindered the OVX‐induced escalation of bone resorption, indicating promising therapeutic prospects for OP. HYP has been shown to decrease phosphorylated Jun N‐terminal kinase (JNK) and p38 levels in osteoblasts in response to H_2_O_2_‐induced dysfunction, thereby providing protection against oxidative damage [15]. Given the documented efficacy of HYP in alleviating oxidative stress and treating OP, it is posited that HYP may ameliorate oxidative damage‐induced OP by attenuating oxidative stress through the regulation of estrogen signaling pathways, as suggested by existing literature.

Dexamethasone (DeX) serves as both an osteoblast‐inducing model drug and a glucocorticoid for the treatment of inflammatory and autoimmune conditions. Prolonged exposure to elevated levels of DeX may result in heightened ROS production within osteoblasts [16]. This study investigates the potential of HYP to inhibit oxidative stress and enhance osteogenic differentiation in osteoblasts and zebrafish through ex vivo and in vivo experiments, drawing upon existing literature. The analysis includes assessment of alkaline phosphatase (ALP) activity, nitric oxide (NO) content, levels of oxidative stress markers such as malondialdehyde (MDA), superoxide dismutase (SOD), and glutathione (GSH), as well as ROS levels, and fish bone formation in zebrafish.A comprehensive methodology involving network pharmacology, molecular docking, and molecular dynamics simulation was employed to study the principal targets and signaling pathways of HYP in the suppression of oxidative stress and treatment of OP. Additionally, RT‐qPCR analysis of target gene proteins was conducted to further elucidate the underlying mechanism of action of HYP in mitigating oxidative stress and managing OP.

## 2 Materials and Methods

### 2.1 Chemicals and Reagents

HYP (purity ≥ 98%) was purchased from Shanghai Ye Biotechnology Co. Ltd. (Batch No. Y26M9X5934), MEM medium (Batch No. 11965092), foetal bovine serum (Batch No. 12483020), penicillin (100 U/mL), and streptomycin (100 μg/mL) (Batch No. 15140122) were purchased from GIBCO (New York, NY, USA). Dimethyl sulfoxide (DMSO) (Batch No. 472301‐500ML), dexamethasone (Batch No. D8041), ascorbic acid (Batch No. A103533), beta glycerophosphoric acid (Batch No. G5422‐1), calcium xanthophyll (Batch No. C302986), AAPH,2,2′‐Azobis(2‐methylpropionamidine) dihydrochloride (Batch No. ajci 23234), 2,7‐difluorofluorescein diacetate (DCFH‐DA) (Batch No. D6470), prednisolone (Batch No. P0180), and etidronate disodium (Batch No. B27158) were purchased from Beijing Chemical Factory Co. NO, SOD, GSH, MDA ELISA kits were purchased from Shanghai Enzyme‐linked Biotechnology Co. Primers were purchased from Wuhan Xavier Biotechnology Co. Reverse transcription kit TAKARA, Japan (Batch No. AN7049A). Fish Hatchery Separator (Patent No. CN202321348742.4.).

### 2.2 Cell Culture and Drug Treatments

The mouse osteoblast cell line MC3T3‐E1 obtained from the Shanghai Cell Bank of the Chinese Academy of Sciences was cultured in MEM‐α medium supplemented with 10% fetal bovine serum,10 U/mL penicillin, and 100 mg/mL streptomycin. Cultivation was conducted at 37°C in a 5% CO_2_ incubator, and cells were passaged at a 1:2 ratio upon reaching 90% confluence. Experimental procedures were initiated during the logarithmic growth phase of the cells. Cell experiments were divided into four groups: the control group receiving a complete medium, the model group treated with 100 μM dexamethasone, the osteoblast‐positive group, oxidative stress‐positive group receiving 10 μM Vitamin D3, 50 μM Vitamin C, and the drug group where dexamethasone modeling was followed by the addition of varying concentrations of HYP (1, 2.5, 5, 10, 20, 40, and 80 μM).

### 2.3 Measurement of Cell Viability

MC3T3‐E1 cells in the logarithmic growth phase were seeded at a density of 5 × 10^3^ cells per well in 96‐well plates and incubated in a 5% CO_2_ atmosphere at 37°C for 24 h to allow for cell adhesion, with the exception of the control group. Subsequently, 200 μL of 100 μM DeX was added to each well. Following a 24‐h incubation period, varying concentrations of HYP were introduced to the cell culture. After an additional 24 h, the culture medium was removed, and 100 μL of a 10% CCK8 solution was added to each well, followed by a 1‐h incubation period. The orange‐yellow liquid was detected at a wavelength of 450 nm, and the survival rate was determined by calculating the percentage of cell survival using the formula:

Cellsurvival%=(ODvalueofdruggroup/ODvalueofcontrolgroup)×100%.

### 2.4 Intracellular ROS Assay

Cell groups in Section 2.2 were treated with a replacement of 10 μM Vitamin C in the positive group. Following a 24‐h incubation period, the cells were exposed to the fluorescent probe 2,7‐dichlorodihydrofluorescein diacetate 10 μg/mL (DCFH‐DA) for 1 h under light protection. Subsequently, the cells were washed three times with phosphate buffer solution (PBS), the extracellular DCFH‐DA solution was removed, and the cells were visualized and photographed using a fluorescence microscope (×10) with excitation at 485 nm and emission at 538 nm.

### 2.5 Osteoblast Differentiation Assay

MC3T3‐E1 cells were cultured in six‐well plates at a density of 1 × 10^4^ cells per well, with the osteogenic induction medium being replaced when the cells approached confluence. The osteogenic induction medium consisted of 10% serum MEM‐α medium, 1% ascorbic acid (5 g/L),1% sodium β‐glycerophosphate (1 mol/L), and 0.1% DeX (10 μM). Following this, varying concentrations of HYP (5, 10, and 20 μM) were introduced for intervention, with the osteogenic induction medium being refreshed every 3 days. After 7 days of incubation, the induction medium was removed. The cells were subjected to two washes with phosphate‐buffered saline (PBS), followed by fixation with 4% paraformaldehyde for a duration of 10 min. Subsequently, incubate in accordance with ALP staining solution (BCIP/NBT working solution) for 1 h away from light. Upon the appearance of a blue precipitate at the base of the well plate, the stain was discarded, and PBS was added to halt the cellular reaction. The cells were then observed under a microscope and images were captured.

### 2.6 Detection of ALP, SOD, NO, GSH, and MDA Content by HYP

The experiment utilized ALP as a model, wherein the cell supernatant was subjected to incubation with either 1 mg/mL of p‐nitrophenyl phosphate (pNPP) as a substrate or substrate‐free conditioned medium (utilized as a control) for approximately 1 h. SOD, NO, GSH, and MDA levels were quantified following the manufacturer's instructions of the respective kits, and the absorbance at 405 nm was assessed via zymography.

### 2.7 Zebrafish Feeding and Breeding

Healthy adult wild‐type AB strain zebrafish obtained from Nanjing Yishu Lihua Biotechnology Co. Ltd. were housed in a recirculating aquatic habitat system with a photoperiod of 14 h of light and 10 h of darkness. Salinity levels were maintained within the range of 0.03%–0.04%. Breeding was conducted using a ratio of one males to two females.

The implementation of a small fish hatchery separator featuring a separating screen within the tank chamber effectively divides the space into distinct upper and lower zones for zebrafish mating and egg deposition. Each gender is provided with designated activity and mating areas, with connectivity facilitated through a connecting channel equipped with a lifting gate. Upon reaching the predetermined start time, the lifting gate opens to enable interconnection between the activity areas of the females and males, facilitating successful mating and spawning processes. Fertilized zebrafish eggs were retrieved and transferred to Petri dishes for incubation in a biochemical incubator set at 28.5°C. Throughout the incubation period, routine monitoring was conducted to remove contaminants and deceased embryos, as well as to replace the embryo water. The zebrafish were fed a diet of dry food or brine shrimp three times daily. All zebrafish‐related procedures were conducted in accordance with the guidelines and approval of the Institutional Committee on Animal Ethics and Use at Changchun University of Traditional Chinese Medicine (No. 2021519).

### 2.8 In Vivo Toxicity Experiments in Zebrafish

One hundred fertilized embryos of wild‐type AB strain zebrafish were collected at 3 days post‐fertilization (3dpf) and randomly allocated into groups with varying concentrations of HYP, prednisolone, and AAPH. The concentrations of HYP (5, 10, 20, 40, and 80 mM), prednisolone (10, 15, 20, 25, and 30 μM), and AAPH (10, 15, 20, 25, and 30 mM) were administered to each group to assess their toxicity on zebrafish embryos.

Survivalrate=(survivingembryosat72hpf/totalembryos)×100%.

### 2.9 Detection of ROS in Zebrafish

Zebrafish embryos fertilized at 7–9 h post‐fertilization (hpf) were randomly chosen and placed in groups of 10 embryos per well in 24‐well plates. The embryos were then immersed in embryo medium, with three replicate wells established for each group to assess the solubilization of zebrafish embryo fetal water. The experimental procedure involved treating zebrafish with a concentration of 20 mM AAPH to induce oxidative stress, with the experimental groups consisting of a control group, model group, drug group, and a positive group treated with Vitamin C. The drug group was administered varying concentrations of HYP (5, 10, and 20 mM). Following embryo development to fertilization, zebrafish larvae were exposed to a 20 μg/mL DCFH‐DA fluorescent ROS probe in a light‐free environment and incubated for 1 h in embryo culture medium, anesthetized, and examined using a fluorescence inverted microscope, with the fluorescence intensity being analyzed using Image Pro Plus 6.0 software.

### 2.10 Calcium Xanthophyll Labeling and Bone Formation Calculations in Zebrafish

Zebrafish embryos of the wild‐type AB strain were randomly allocated into four experimental groups: control, model, positive drug, and drug. The drug group consisted of five concentrations (5, 10, 20, 40, and 80 mM) with five embryos in each concentration group. Following fertilization, the embryos were allowed to develop until 3dpf, at which point prednisolone, the modeling drug, was administered for a duration of 2 days, excluding the control group. Zebrafish larvae were subjected to staining with a 2% calcium xanthophyll marker, followed by anesthesia and triple rinsing with embryonic water. Subsequently, the larvae were observed and photographed using a fluorescence microscope to detect osteocalcin in the vertebral region within the wavelength range of 510–550 nm. The extent of calcium xanthophyll staining in the spinal skeleton of the larvae was quantified, and the cumulative optical density was analyzed quantitatively using Image Pro Plus 6.0 image analysis software.

### 2.11 Network Pharmacological Analysis

The HYP target was screened using Swiss Target Prediction and Pharm Mapper databases, while the OP target was screened using Gene Gards, OMIM, and TTD databases with the keywords “osteoporosis” and “oxidative stress.” The targets were imported into Cytoscape 3.8.0 for visualization and analysis. Core targets were selected based on high Betweenness Centrality, Closeness Centrality, and Degree values compared to the average, and a PPI protein interaction network was constructed. Enrichment analyses were conducted using the Metascape database for GO and KEGG pathways. Data visualization was done using the Microbiotics platform with GO bubble charts, KEGG enrichment bubble charts, and GO triple bar charts.

### 2.12 Molecular Docking and Molecular Dynamics Simulations

Molecular docking of the core targets AKT (PDBID: 6NPZ), ESR1 (PDBID: 6VPF), IGF1 (PDBID: 4XLV), PPARG (PDBID: 8B8W), SRC (PDBID: 3DQX), The receptor proteins used for molecular docking were obtained from the UniProt and RCSB PDB databases and saved in “pdb” format. The 3D chemical structure of HYP was obtained from PubChem and converted using Openbabel 2.4.1 software. The file is then saved as a ligand file in “pdbqt” format. Automated generation of docking sites using SailVina based on the original ligands in the downloaded receptor proteins [17]. Autodock Vina was then used to dock HYP to the target molecules, with binding energies below −5.0 kcal/mol indicating good binding activity. Results were visualized and analyzed using PyMOL software.

Molecular dynamics simulations confirmed HYP's stability when binding to the target protein using a 200 ns simulation with a CHARMm force field. The structural stability was assessed through the root mean square deviation (RMSD), the root mean square fluctuation (RMSF), and radius of gyration (RG) calculations from the simulation trajectories. Throughout the simulation, low and smooth RMSD values indicated a tight binding between the ligand and receptor, resulting in increased stability of the complex.

### 2.13 RNA Isolation and RT‐qPCR

The MC3T3‐E1 cell experimental groups during logarithmic growth included a blank control group, a 100 μM DeX group, a 5 μM HYP administration group, and a 10 μM Vitamin D_3_ positive drug group. After 24 h of treatment, RNA was extracted, its concentration determined, and the necessary amount calculated for genomic DNA removal, reverse transcription, and PCR amplification. The amplification conditions included a pre‐denaturation step at 90°C for 15 s, followed by annealing at 60°C for 1 min, and a final denaturation step at 90°C for 15 s, repeated for a total of 40 cycles. GAPDH was utilized as an internal reference for both the procedure and gene normalization, with relative quantitative analysis conducted using the 2^−ΔΔCt^ method. The specific primer sequences can be found in Table 1.

**Table 1:** Sequences of RT‐qPCR primers.

Gene name	Sequence (5′–3′)	Primer length
GAPDH	CCTCGTCCCGTAGACAAAATG TGAGGTCAATGAAGGGGTCGT	133
SRC	AGATCACTAGACGGGAATCAGAGC GCACCTTTTGTGGTCTCACTCTC	94
IGF1	GACCGCACCTGCAATAAAGATAC CCTGTGGGCTTGTTGAAGTAAA	183
AKT1	CTTCCTCCTCAAGAACGATGGC TGTCTTCATCAGCTGGCATTGT	118
PPARG	TGGAGCCTAAGTTTGAGTTTGC GGGGTGAAGGCTCATGTCTG	311
PI3K	CAAACCACCCAAGCCCACTA AGGTCCCATCAGCAGTGTCTC	137
ESR1	TGTTGGATGCTGAACCGCC CCAGACGAGACCAATCATCAGA	224
eNOS	CTGCCACCTGATCCTAACTTGC AGCCCTTTGATCTCAATGTCGT	110
FOXO	GTGTTTGGACCTTCGTCTCTGA GAGTGTCTGGTTGCCGTAGTGT	111

### 2.14 Statistical Analysis

Each group underwent three parallel experiments, with results presented as mean ± SD. Statistical comparisons were conducted using one‐way ANOVA in SPSS 20.0, with significance set at *p* < 0.05.

## 3 Results

### 3.1 Effect of HYP on Osteoblast Viability

**Figure 1:**
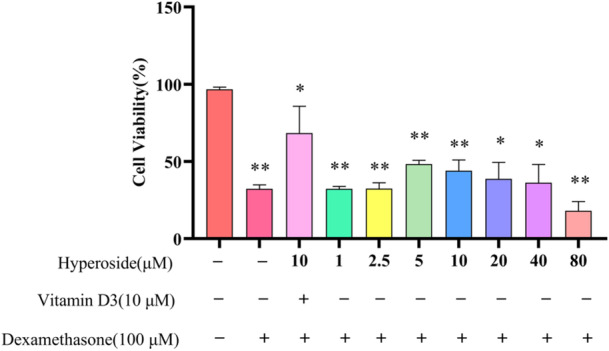
MC3T3‐E1 cell viability (data is presented as means ± SD) (*n* = 3). Compared to the control group, **p < 0.01, *p < 0.05.

As depicted in Figure 1, the cell survival rate exhibited significant variation across different concentrations of HYP. The concentrations of HYP ranged from 1 to 80 μM, resulting in varying degrees of cell growth promotion. However, 1 μM, 2.5 μM of HYP resulted in lower survival of osteoblasts after injury, and 80 μM of HYP prevented cell survival. Notably, the survival rates of cells treated with 5, 10, and 20 μM were comparatively higher, leading to the selection of these concentrations as the basis for subsequent ROS and ALP staining experiments.

### 3.2 Effects of Oxidative Stress on HYP Osteoblasts

**Figure 2:**
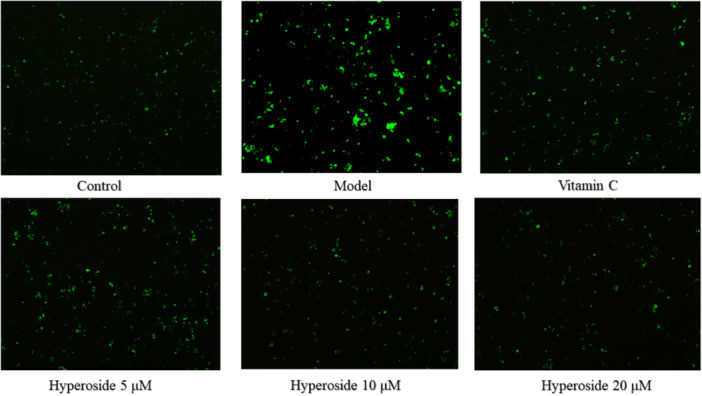
Fluorescence staining results of ROS in MC3T3‐E1 cells.

The DCFH‐DA fluorescent probe demonstrates a reaction with intracellular ROS, resulting in green fluorescence. The intensity of this fluorescence is directly proportional to the concentration of ROS present. Analysis of the fluorescence intensity across different experimental groups, as depicted in Figure 2, revealed the highest intensity in the model group, confirming the successful establishment of the model. In comparison to the control group, the HYP administration group exhibited a concentration‐dependent increase in fluorescence area, resulting in a reduction in ROS generation. This suggests that HYP has a significant impact on the inhibition of oxidative stress in MC3T3‐E1 cells. Based on assessments of osteoblast survival rate and ROS fluorescence intensity,a concentration of 20 μM of HYP demonstrated the most favorable effect.

### 3.3 Cellular ALP Staining

**Figure 3:**
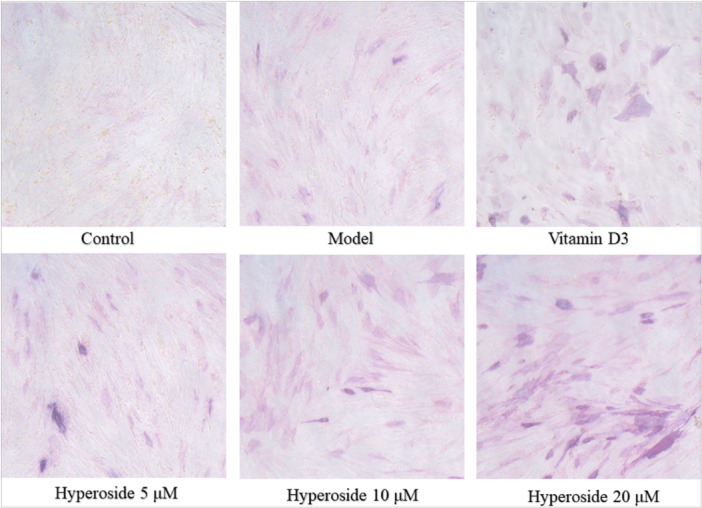
Alkaline phosphatase stain.

Stained using the ALP kit, ALP serves as a crucial marker for early osteoblast differentiation. The intensity of the coloration is directly proportional to the presence of ALP. As depicted in Figure 3, minimal color change was observed in the control group, while the model group exhibited a darker hue, suggesting successful modeling. Various concentrations of HYP resulted in varying color changes, with 20 μM HYP exhibiting the darkest color, suggesting that it was the most effective concentration for stimulating differentiation of MC3T3‐E1 cells. Concentrations of 5 and 10 μM also facilitated differentiation of MC3T3‐E1 cells, albeit with slightly lower levels of ALP compared to 20 μM HYP.

### 3.4 Effect of HYP on ALP, SOD, NO, GSH, and MDA

**Figure 4:**
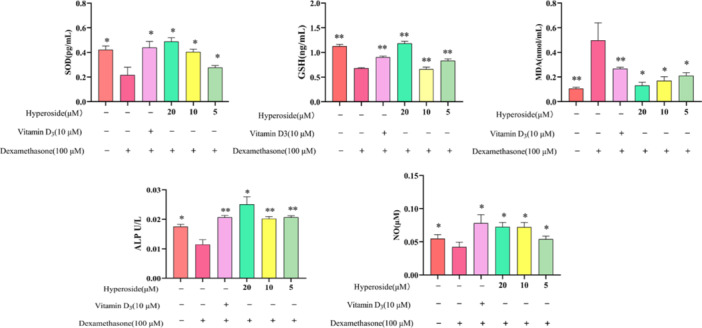
SOD, GSH, MDA, ALP, NO quantity contained (data is presented as means ± SD) (*n* = 3). Compared to the model group, **p < 0.01 and *p < 0.05.

The levels of ALP, SOD, NO, GSH, and MDA in the cells were quantified using a specific kit, as depicted in Figure 4. A comparison of these levels among different groups revealed statistically significant differences (*p* < 0.05) as determined by ANOVA. In comparison to the control group, the model group exhibited lower ALP content in cells when compared to the HYP‐administered group, indicating a notable impact of HYP on osteogenic differentiation. Furthermore, the HYP group demonstrated a decrease in MDA levels and an increase in NO, GSH, and SOD levels in a dose‐dependent manner when compared to the model group, suggesting the ability of HYP to mitigate cellular oxidative stress damage.

### 3.5 HYP Prednisolone and AAPH Toxicity Concentration Study

**Figure 5:**
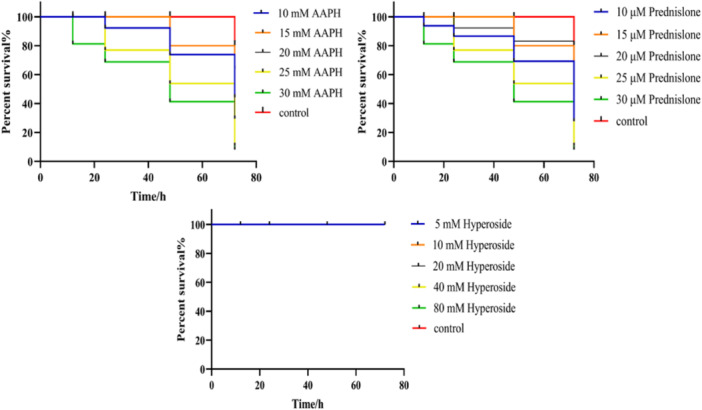
Survival of zebrafish by AAPH, prednisolone, and HYP.

The graphical representation in Figure 5 illustrates the influence of different concentrations of HYP on the survival rate of zebrafish, showing minimal toxicity at all levels. The results suggest that the survival of zebrafish larvae was affected by each experimental concentration of prednisolone and AAPH, with a reduction in survival rate correlating with the dose of prednisolone. Based on the literature, the concentrations of prednisolone at 15 μM and AAPH at 15 mM were finally determined.

### 3.6 Effects of Zebrafish on Oxidative Stress in Juvenile Fish

**Figure 6:**
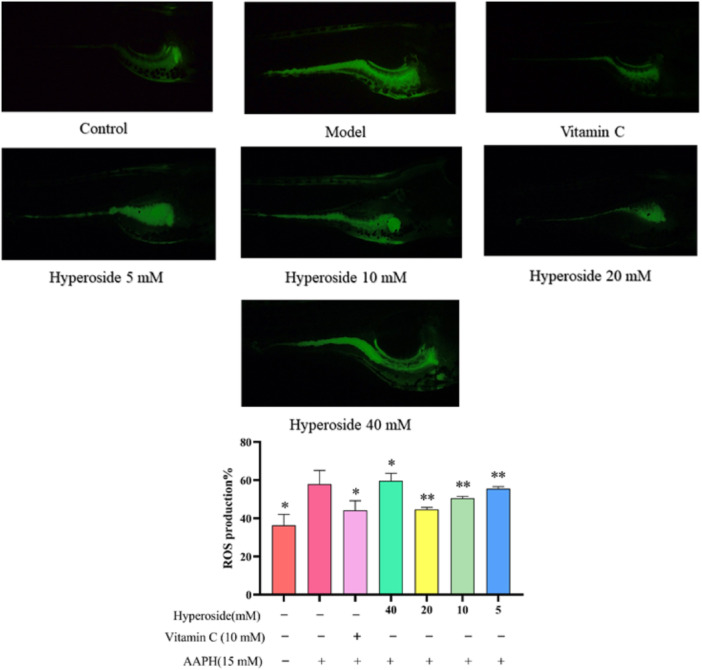
Effects of HYP on oxidative stress in zebrafish (data is presented as means ± SD) (*n* = 3). Compared to the model group, **p < 0.01 and *p < 0.05.

As illustrated in Figure 6, the fluorescence area of zebrafish induced by AAPH exhibited a notable increase relative to that of the control group, implying the potential of AAPH to promote the generation of ROS in zebrafish embryos. In contrast, the fluorescence area of the group treated with HYP was observed to decrease compared to the model group, indicating a reduction in ROS levels and suggesting that HYP exerts an inhibitory influence on ROS in zebrafish larvae.

### 3.7 Zebrafish Bone Formation

**Figure 7:**
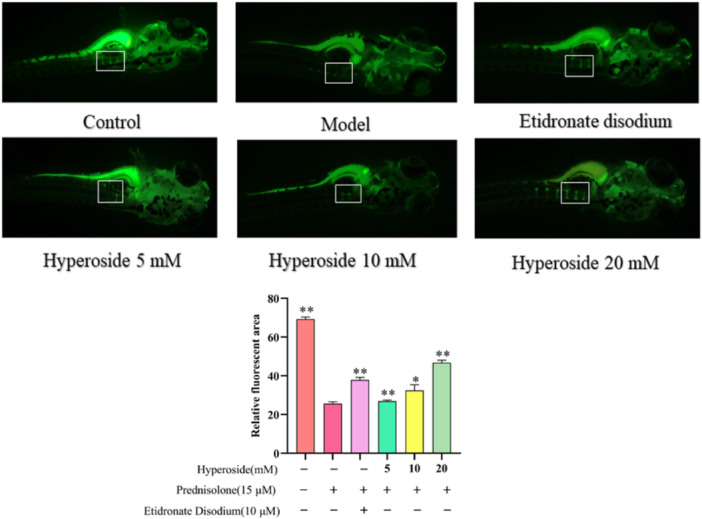
Effects of HYP on bone formation in zebrafish (data is presented as means ± SD) (*n* = 3). Compared to the model group, **p < 0.01 and *p < 0.05.

Based on the results of the Section 2.10 zebrafish toxicity test, Furthermore, the model of prednisolone‐induced OP in zebrafish was successfully established after a 3‐day administration period. As illustrated in Figure 7, the relative fluorescence area of the fish vertebrae was significantly lower in comparison to both the model group and the blank control group, suggesting successful experimental modeling of zebrafish. Similarly, upon comparing the experimental HYP group with the model group, it was observed that the relative fluorescence area of the HYP group was significantly greater than that of the model group. Furthermore, the relative fluorescence area of the HYP group exceeded that of the control group, suggesting that HYP facilitates bone formation in zebrafish.

### 3.8 Network Pharmacological Analyses

**Figure 8:**
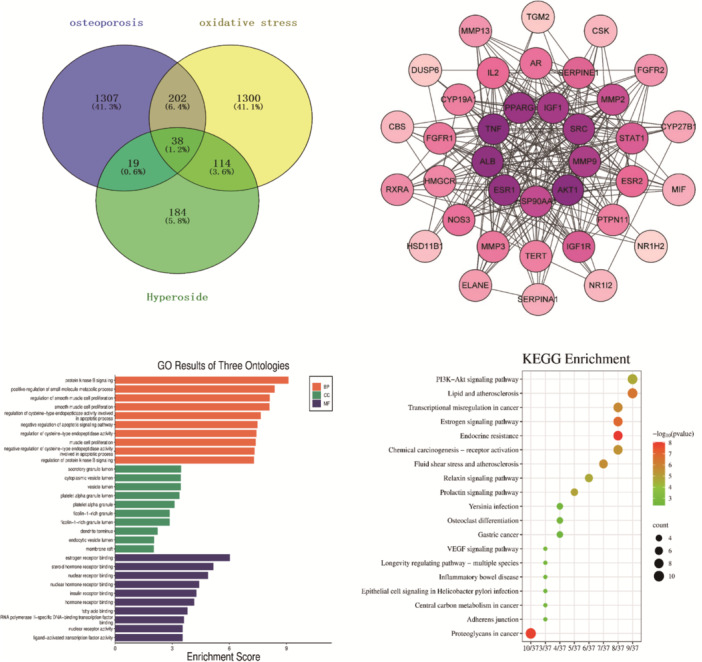
PPI, GO, KEGG analysis.

The Swiss Target Prediction and Pharm Mapper databases were utilized for the screening of 335 HYP targets. Furthermore, the Gene Gards, OMIM, and TTD disease databases were employed to identify disease targets associated with “osteoporosis” and “oxidative stress”, resulting in the discovery of 1566 OP targets, 1645 oxidative stress targets, and 38 targets that were common to both categories. Venny was utilized as depicted in Figure 8 and imported into Cytoscape 3.8.0 for visualization and analysis. The Betweenness Centrality value, Closeness Centrality value, and Degree value, exceeding the average, were employed to identify nine core targets and establish the PPI protein interaction network. The Metascape database was utilized for conducting GO and KEGG pathway enrichment analyses. The data was visualized using a microbiology platform on May 9, 2023, and presented as bubble plots and bar charts. KEGG enrichment analysis identified 38 signaling pathways, with the top 20 pathways selected based on ‐logP value (Figure 8).

### 3.9 Molecular Docking and Molecular Dynamics Simulations

**Figure 9:**
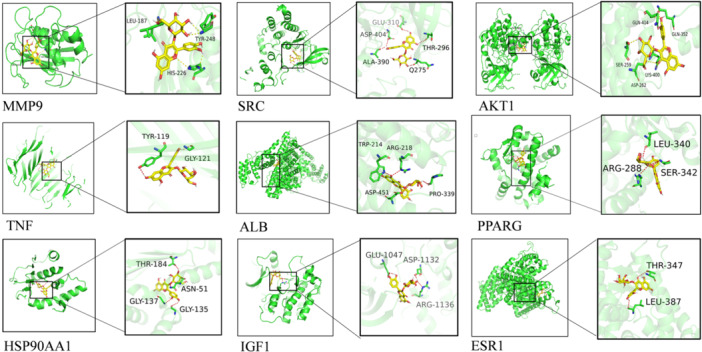
Molecular docking.

**Figure 10:**
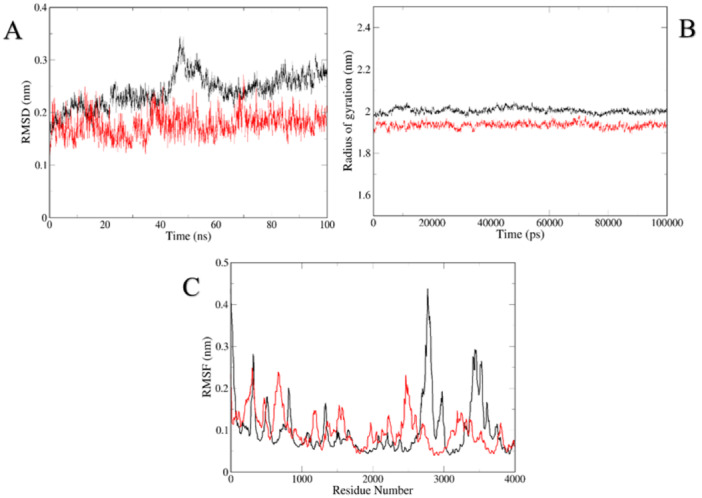
Molecular docking and molecular dynamics simulation. (A) RMSD analysis, (B) GR analysis, and (C) RMSF analysis.

The binding energies of HYP to nine core targets were determined through computational analysis using Auto Dock Vina software. The calculated docking binding energy values for HSP9AA1, MMP9, ALB, IGF1, SRC, AKT1, TN, FPPARG, ESR1, and HYP were −9.4, −8.8, −8.3, −7.8, −7.8, −7.5, −7.5, −7.1, and −6.3 kcal/mol, respectively (Figure 9). Overall, binding energies below −5.0 kcal/mol suggest strong binding activity of the molecules to the targets, as observed through visualization with PyMOL software. To assess the stability of the protein target in the presence of HYP, molecular dynamics simulations were conducted, revealing insights into the behavior of the system over the simulation period, as depicted in Figure 10. The RMSD values consistently maintained a low range, typically between 0.2 and 0.3. Analysis of the RMSF plot indicates that the majority of receptor residues interacting with HYP via AKT1 and SRC remain within a stable range. Additionally, the decreased RG of the proteins suggests a more compact structure and stable complexes.

### 3.10 Results of RT‐qPCR Experiments

**Figure 11:**
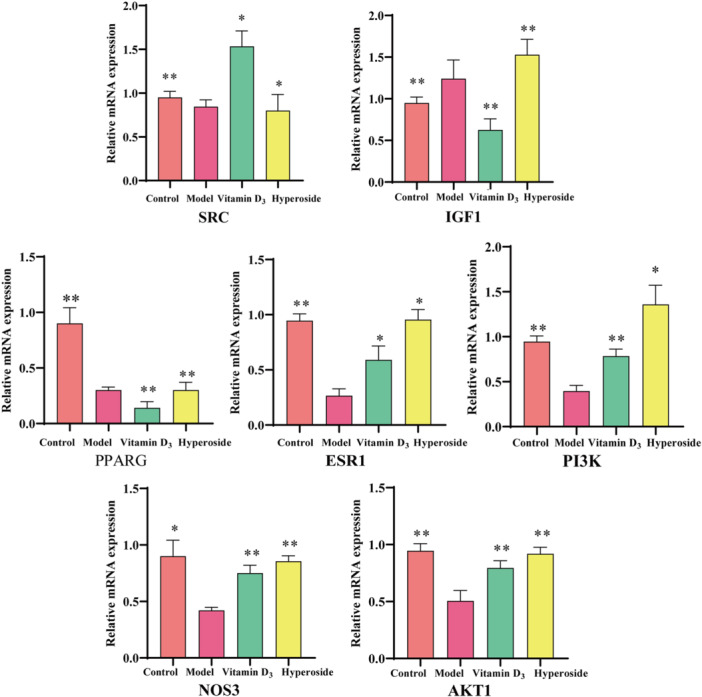
RT‐qPCR analysis shows HYP's impact on the mRNA expression levels of core target genes. Gene expression was normalized to GAPDH the 2^−∆∆CT^ method (data is represented as the mean ± SD) (*n* = 3). Compared with model, **p < 0.01, *p < 0.05.

To further assess the cellular activity of HYP at the gene level, RT‐qPCR was employed, revealing significantly distinct mRNA levels between the model group and the control group (*p* < 0.01), indicating successful modeling. In comparison to the control group, the mRNA levels of AKT1, SRC, ESR1, PI3K, and NOS3 were notably increased following administration of HYP (*p* < 0.05), while IGF1 and PPARG exhibited a downward trend, all of which were statistically significant (Figure 11).

## 4 Discussion

As the population continues to age, OP has emerged as a significant public health concern necessitating early prevention and treatment to preserve the equilibrium between osteoblasts and osteoclasts and prevent fractures. Consequently, the exploration of natural and efficacious anti‐OP medications is imperative [18, 19].

The established role of HYP in mitigating oxidative stress and facilitating osteogenic differentiation is well‐documented, however, the underlying mechanism of action remains unclear [20, 21]. This study demonstrates that HYP promotes osteoblast proliferation within the concentration range of 5–20 μM. To further elucidate the impact of HYP on oxidative stress, the ROS levels in osteoblasts following oxidative damage were evaluated using a ROS fluorescent probe (DCFH‐DA). The fluorescence intensity of the HYP administration group was found to be lower than that of the model group, suggesting its potential to mitigate oxidative damage in osteoblasts. The analysis of osteoblast supernatant demonstrated a significant increase in levels of SOD, GSH, and NO, as well as a decrease in MDA levels compared to the model group. These findings suggest that HYP induces the production of antioxidants and anti‐free radicals in osteoblasts that have been induced by Dex. SOD and GSH enzymes with direct free radical scavenging capabilities, serve as abundant intracellular antioxidants that can effectively mitigate oxidative stress [22, 23]. Conversely, MDA functions as a highly reactive metabolite indicative of oxidative stress, generated through the oxidation of free radicals and other oxidative stress‐inducing agents within the body. The aforementioned findings collectively indicate that HYP exhibits therapeutic properties in mitigating oxidative damage in osteoblasts. The upregulation of ALP serves as a crucial marker for osteoblast function and bone health. Treatment with HYP led to enhanced ALP activity, facilitating early osteoblast differentiation [24]. Furthermore, in vivo studies using the zebrafish model confirmed the protective effects of HYP against oxidative stress and OP. Zebrafish, particularly in the embryonic stage, serve as a commonly utilized model for investigating the development of osteoblasts due to the similarity of their bone structure to that of humans [25]. Through in vivo experiments involving zebrafish, it was observed that HYP mitigated oxidative damage in a model of AAPH‐induced oxidative stress, indicating its potential as an inhibitor of oxidative stress and a protective agent against both oxidative damage and OP. Moreover, HYP was found to enhance the area of prednisolone‐induced skeletal fluorescence and facilitate skeletal development in zebrafish larvae. The results of both in vivo and ex vivo experiments indicate that HYP has the potential to ameliorate OP through the inhibition of oxidative stress.

To enhance understanding of the therapeutic mechanism of HYP in treating OP, a methodology combining network pharmacology, molecular docking, and molecular dynamics was employed. Through the identification of key targets associated with oxidative stress in OP and HYP components, it was determined that HYP exerts its effects by targeting 38 specific proteins. Subsequent protein–protein interaction analysis revealed AKT1, PPARG, ESR1, IGF1, SRC, ALB, TNF, HSP9AA1, and MMP9 as central targets in the mechanism of action of HYP. SRC is a component of the Src family of kinases (SFK) involved in the regulation of bone metabolism homeostasis [26, 27]. In osteoblasts, SRC functions by activating YAP and STAT1 to suppress Runx2 transcription and osteoblast differentiation, making it a promising target for OP treatment. Additionally, simultaneous modulation of SRC has been shown to mitigate oxidative stress. In osteoclasts, Kisspeptin‐10 binds to Gpr54 to prevent bone loss by activating the dephosphorylation of Src mediated by dusp18 [28]. Zhao Fang et al. [29] demonstrated that inhibition of the PI3K/AKT pathway can reduce oxidative stress, inflammation, and apoptosis in cardiac remodeling [30]. The literature suggests that PI3K plays a role in mitigating bone loss induced by oophorectomy through the regulation of lipid metabolism [31]. By activating PI3K/AKT signaling pathway in osteoblasts, it was shown that simiao wan promoted osteoblast proliferation, ALP activity, calcium nodule deposition and the expression of osteogenic markers (osteocalcin [cyanate], Runt‐related transcription factor 2 [RunX2] and Type I collagen) [32]. If PI3K is directly activated in osteoblasts and osteoblasts, the sex‐specific effect on the cortical surface can enhance the bone of mice [33]. Additionally, AKT has been reported to be involved in the regulation of cell proliferation, differentiation, and apoptosis, with phosphorylation of DlX3 by AKT leading to increased protein stability [34], osteogenic activity, and transcriptional activity of DlX3. NOS3, an important regulatory enzyme, is activated to catalyze l‐arginine in endothelial cells, producing NO with vasodilatory effects and significantly reducing levels of ROS. Therefore, the promotion of local blood supply in blood vessels and enhancement of microcirculation in small blood vessels within bone are crucial factors in regulating osteoclast and osteoblast differentiation [35]. Additionally, studies have shown that post‐translational modification of PPARG plays a significant role in regulating bone formation and resorption [36]. When the mouse model of bone cell‐specific PPARG is knocked out, it is proved that PPARG controls the biological energy metabolism of bone cells and its contribution to the whole body energy metabolism, regardless of the level of circulating sclerosis protein, especially PPARG acts as a molecular barrier of mitochondrial function in male bone cells, protecting them from oxidative stress and ROS accumulation [37]. Furthermore, research conducted by Xinghui Wang et al. [38] demonstrated that propionate can mitigate fatty acid‐induced mitochondrial dysfunction and oxidative stress‐induced apoptosis by upregulating PPARG co‐activator 1α in hepatocytes [38]. Weiwei Li [39] and colleagues discovered that increasing the expression of IGF1 in human ovarian granulosa cells activates the PI3K/Akt signaling pathway, leading to a reduction in hydrogen peroxide‐induced oxidative stress and apoptosis. Additionally, IGF1, a prominent growth factor found in bone matrix, is intricately linked to bone growth and metabolism [40]. Furthermore, ESR1 has the ability to promote osteoblast differentiation in an estrogen receptor‐dependent manner, thereby exerting a protective effect against OP [41]. Coumarin‐derived Umbrella (UF) significantly improves OP patients through downstream activation of estrogen receptor 1 (ESR1) and β‐catenin pathway [42]. Consequently, these key targets demonstrate efficacy in combating oxidative damage and preventing OP.

**Figure 12:**
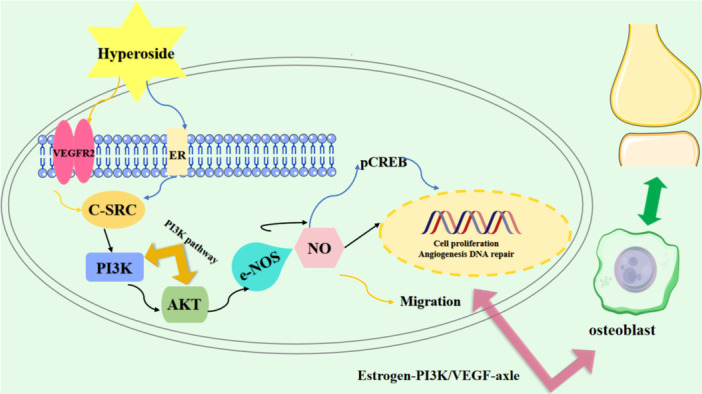
Mechanism of the estrogen‐PI3K/VEGF signaling pathway axis map.

By enrichment analysis of the intersecting targets of HYP, oxidative stress and OP, the KEGG results indicated that the estrogen signaling pathway, PI3K signaling pathway, and vascular endothelial growth factor (VEGF) signaling pathway were the relevant pathways of HYP for anti‐OP and amelioration of oxidative stress (Figure 12). The estrogen signaling pathway is mainly mediated by estrogen, and the formation of reactive metabolites on the estrogen receptor activates antioxidant signaling by estrogen action [43]. It can also be involved in generating anti‐OP effects by mediating multiple signaling pathways such as WNT, BMP‐2/Smad, AKT/ERT [44, 45]. Activation of the PI3K/AKT signaling pathway has been shown to promote osteogenic differentiation and modulate protein expression levels to mitigate oxidative stress [46]. Additionally, research has demonstrated the physiological necessity of VEGF in regulating endothelial cell proliferation during angiogenesis and in maintaining vascular integrity throughout the lifespan of blood vessels. Pathological states, such as oxidative stress, have been shown to increase the expression of VEGF, a key player in the signaling pathway that is crucial for bone healing and reconstruction [47, 48]. Additionally, the transcription factor RUNX2 has the ability to activate VEGF, stimulate angiogenesis, improve the bone microenvironment, replenish microvessels within the bone, thereby safeguarding against bone loss and promote bone growth [49, 50]. By examining the regulatory effects of key proteins in the pathway on upstream and downstream targets. The results showed that HYP promoted the up‐regulation of AKT1, SRC, NOS3, PI3K and ESR1 targets, promoted NO production, inhibited the down‐regulation of PPARG and IGF1 targets, inhibited oxidative stress, promoted bone formation and improved OP. Ultimately, molecular docking and molecular dynamics simulation techniques were employed to assess the stability of HYP for key proteins within the estrogen‐PI3K/VEGF axis, successfully corroborating experimental findings through comprehensive computer simulations from various angles. Hence, based on the findings of the experiment, it is hypothesized that the estrogen‐PI3K/VEGF signaling axis functions to suppress oxidative stress and enhance the OP pathway, leading to elevated levels of NO, decreased ROS, mitigation of intracellular oxidative damage, ultimately facilitating bone formation and ameliorating OP.

This study recognizes specific limitations that will be rectified in future research efforts. For example, gene expression was not assessed in the in vivo zebrafish experiments, and the genes of interest are not clearly defined in vivo. Additionally, only osteoblast cell differentiation was investigated, which does not comprehensively explain the mechanism of HYP's resistance to OP by inhibiting oxidative stress research mechanisms. In the subsequent investigation, the research team will conduct further examination of osteoblast precursor cells, specifically bone marrow mesenchymal stem cells (BMSC), in order to delve deeper into the inhibition of oxidative stress within the estrogen‐PI3K/VEGF axis mechanism for enhancing OP treatment.

## 5 Conclusion

This study explores the potential of HYP to activate the estrogen‐PI3K/VEGF axis, thereby suppressing oxidative stress and enhancing osteogenic differentiation to mitigate OP. The findings suggest that HYP plays a crucial role in addressing oxidative stress‐induced OP, and its mechanism of action involves the modulation of the estrogen‐PI3K/VEGF signaling pathway. These results offer a novel therapeutic approach for combating OP in the future.

## Author Contributions

**Shuo Wang:** conceptualization. **Wei Feng:** conceptualization. **Xueqin Feng:** software. **Peitong Wu:** validation. **Nanxi Zhang:** methodology. **Xiaoqian Yang:** visualization. **Yawen Li:** investigation. **Chunnan Li:** funding acquisition. **Jiaming Sun:** supervision.

## Ethics Statement

The authors agree to be accountable for all aspects of the work to ensure that questions related to the accuracy or integrity of any part of the work are appropriately investigated and resolved. The animal study protocol was approved by the Laboratory Animal Ethics Committee of Changchun University of Chinese Medicine (Protocol Code: 2021519, date of approval: 19 November 2021) for studies involving animals.

## Conflicts of Interest

The authors declare no conflicts of interest.

## Data Availability

The original contributions presented in the research are included in the manuscript, and further queries can be engaged to the corresponding authors. All data are not in public databases and are available on request.

## References

[1] Z. Zhao , M. Nian , H. Lv , et al., “Advances in Anti‐Osteoporosis Polysaccharides Derived From Medicinal Herbs and Other Edible Substances,” American Journal of Chinese Medicine 50 (2022): 441–470, 10.1142/S0192415X22500173. 35021963

[2] J. M. Kim , C. Lin , Z. Stavre , M. B. Greenblatt , and J. H. Shim , “Osteoblast‐Osteoclast Communication and Bone Homeostasis,” Cells 9 (2020): 2073, 10.3390/cells9092073. 32927921 PMC7564526

[3] H. Chen , Z. Weng , M. Kalinowska , et al., “Anti‐Osteoporosis Effect of Bioactives in Edible Medicinal Plants: A Comprehensive Review,” Critical Reviews in Food Science and Nutrition 65, no. 22 (2025): 4310–4326, 10.1080/10408398.2024.2386449. 39093554

[4] Q. Liu , M. Li , S. Wang , Z. Xiao , Y. Xiong , and G. Wang , “Recent Advances of Osterix Transcription Factor in Osteoblast Differentiation and Bone Formation,” Frontiers in Cell and Developmental Biology 8 (2020): 601224, 10.3389/fcell.2020.601224. 33384998 PMC7769847

[5] Y. K. Oh , N. H. Moon , and W. C. Shin , “Management of Osteoporosis Medication After Osteoporotic Fracture,” Hip & Pelvis 34 (2022): 191–202, 10.5371/hp.2022.34.4.191. 36601612 PMC9763832

[6] J. Tsai , K. Kaneko , A. J. Suh , R. Bockman , and K. H. Park‐Min , “Origin of Osteoclasts: Osteoclast Precursor Cells,” Journal of Bone Metabolism 30 (2023): 127–140, 10.11005/jbm.2023.30.2.127. 37449346 PMC10346003

[7] Q. Geng , S. Wang , K. Heng , et al., “Astaxanthin Attenuates Irradiation‐Induced Osteoporosis in Mice by Inhibiting Oxidative Stress, Osteocyte Senescence, and SASP,” Food & Function 13 (2022): 11770–11779, 10.1039/d2fo01673g. 36285709

[8] J. Fan , K. Yuan , X. Zhu , S. Cui , H. Yi , and W. Zhang , “Neuroligin‐3 Activates Akt‐Dependent Nrf2 Cascade to Protect Osteoblasts From Oxidative Stress,” Free Radical Biology and Medicine 208 (2023): 807–819, 10.1016/j.freeradbiomed.2023.09.032. 37774803

[9] L. Yu , W. Wang , C. Lv , et al., “Dual Functional Hydrogel of Osteoclastic‐Inhibition and Osteogenic‐Stimulation for Osteoporotic Bone Defect Regeneration,” Mater Today Bio 31 (2025): 101550, 10.1016/j.mtbio.2025.101550. PMC11867540 40018058

[10] S. Xu , S. Chen , W. Xia , H. Sui , and X. Fu , “Hyperoside: A Review of Its Structure, Synthesis, Pharmacology, Pharmacokinetics and Toxicity,” Molecules 27, no. 9 (2022): 3009, 10.3390/molecules27093009. 35566359 PMC9101560

[11] C. Wang , X. Li , Z. Liu , M. L. Han , Y. L. Hou , and C. L. Guo , “[The Effect and Mechanism of Hyperoside on High Glucose‐Induced Oxidative Stress Injury of Myocardial Cells],” Sichuan da xue xue bao. Yi xue ban = Journal of Sichuan University. Medical Science Edition 49 (2018): 518–523. 30378302

[12] P. D. S. M. Fernando , M. J. Piao , H. M. U. L. Herath , et al., “Hyperoside Reduced Particulate Matter 2.5‐induced Endoplasmic Reticulum Stress and Senescence in Skin Cells,” Toxicology In Vitro 99 (2024): 105870, 10.1016/j.tiv.2024.105870. 38848825

[13] S. H. Kwon , S. R. Lee , Y. J. Park , et al., “Suppression of 6‐Hydroxydopamine‐Induced Oxidative Stress by Hyperoside via Activation of Nrf2/HO‐1 Signaling in Dopaminergic Neurons,” International Journal of Molecular Sciences 20 (2019): 5832, 10.3390/ijms20235832. 31757050 PMC6929192

[14] Y. Chen , F. Dai , Y. He , et al., “Beneficial Effects of Hyperoside on Bone Metabolism in Ovariectomized Mice,” Biomedicine & Pharmacotherapy 107 (2018): 1175–1182, 10.1016/j.biopha.2018.08.069. 30257331

[15] X. C. Qi , B. Li , W. L. Wu , H. C. Liu , and Y. P. Jiang , “Protective Effect of Hyperoside Against Hydrogen Peroxide‐Induced Dysfunction and Oxidative Stress in Osteoblastic MC3T3‐E1 Cells,” Artificial Cells, Nanomedicine, and Biotechnology 48 (2020): 377–383, 10.1080/21691401.2019.1709851. 31903787

[16] X. Zhang , R. Pang , K. Zhang , et al., “Apocynin Exerts Cytoprotective Effects on Dexamethasone‐Induced Osteoblasts by Inhibiting Oxidative Stress Through the Nrf2 Signalling Pathway,” Journal of Cellular and Molecular Medicine 27 (2023): 3911–3927, 10.1111/jcmm.17974. 37749949 PMC10718140

[17] W. P. Feinstein and M. Brylinski , “Calculating an Optimal Box Size for Ligand Docking and Virtual Screening Against Experimental and Predicted Binding Pockets,” Journal of Cheminformatics 7 (2015): 18, 10.1186/s13321-015-0067-5. 26082804 PMC4468813

[18] B. L. Clarke , “Economic Costs of Severe Osteoporotic Fractures Continue to Increase at Expense of Refracture,” Journal of Bone and Mineral Research 37 (2022): 1809–1810, 10.1002/jbmr.4713. 36252570

[19] T. Xu , J. Yin , X. Dai , et al., “Cnidii Fructus: A Traditional Chinese Medicine Herb and Source of Antiosteoporotic Drugs,” Phytomedicine 128 (2024): 155375, 10.1016/j.phymed.2024.155375. 38507853

[20] W. Zhu , M. Ge , X. Li , et al., “Hyperoside Attenuates Zearalenone‐Induced Spleen Injury by Suppressing Oxidative Stress and Inhibiting Apoptosis in Mice,” International Immunopharmacology 102 (2022): 108408, 10.1016/j.intimp.2021.108408. 34920313

[21] S. Fan , H. Pan , J. Huang , Z. Lei , and J. Liu , “Hyperoside Exerts Osteoprotective Effect on Dexamethasone‐Induced Osteoblasts by Targeting NADPH Oxidase 4 (NOX4) to Inhibit the Reactive Oxygen Species (ROS) Accumulation and Activate C‐Jun N‐Terminal Kinase (JNK) Pathway,” Bioengineered 13 (2022): 8657–8666, 10.1080/21655979.2022.2054499. 35331079 PMC9161895

[22] C. Zhang , Y. Hu , Y. Yuan , et al., “Liposome‐Embedded SOD Attenuated DSS‐Induced Ulcerative Colitis in Mice by Ameliorating Oxidative Stress and Intestinal Barrier Dysfunction,” Food & Function 14 (2023): 4392–4405, 10.1039/d2fo03312g. 37092895

[23] J. Ou , H. Tao , Q. Bao , et al., “Investigating Oxidative Stress Associated With Myocardial Fibrosis by High‐Fidelity Visualization and Accurate Evaluation of Mitochondrial GSH Levels,” Analytical Chemistry 96 (2024): 4232–4241, 10.1021/acs.analchem.3c05603. 38421725

[24] M. A. Sanchez , B. Felice , L. D. Sappia , S. Lima Moura , M. Martí , and M. I. Pividori , “Osteoblastic Exosomes. A Non‐Destructive Quantitative Approach of Alkaline Phosphatase to Assess Osteoconductive Nanomaterials,” Materials Science and Engineering: C 115 (2020): 110931, 10.1016/j.msec.2020.110931. 32600679

[25] S. Vimalraj , D. Govindarajan , S. Sudhakar , R. Suresh , P. Palanivel , and S. Sekaran , “Chitosan Derived Chito‐Oligosaccharides Promote Osteoblast Differentiation and Offer Anti‐Osteoporotic Potential: Molecular and Morphological Evidence From a Zebrafish Model,” International Journal of Biological Macromolecules 259 (2024): 129250, 10.1016/j.ijbiomac.2024.129250. 38199551

[26] L. Salvadori , M. L. Belladonna , B. Castiglioni , et al., “KYMASIN UP Natural Product Inhibits Osteoclastogenesis and Improves Osteoblast Activity by Modulating Src and p38 MAPK,” Nutrients 14 (2022): 3053, 10.3390/nu14153053. 35893905 PMC9370798

[27] T. Matsubara , K. Yasuda , K. Mizuta , H. Kawaue , and S. Kokabu , “Tyrosine Kinase Src Is a Regulatory Factor of Bone Homeostasis,” International Journal of Molecular Sciences 23 (2022): 5508, 10.3390/ijms23105508. 35628319 PMC9146043

[28] Z. Li , X. Yang , R. Fu , et al., “Kisspeptin‐10 Binding to Gpr54 in Osteoclasts Prevents Bone Loss by Activating Dusp18‐Mediated Dephosphorylation of Src,” Nature Communications 15 (2024): 1300, 10.1038/s41467-024-44852-9. PMC10861593 38346942

[29] Z. Fang , F. Yushanjiang , G. Wang , X. Zheng , and X. Jiang , “Germacrone Mitigates Cardiac Remodeling by Regulating PI3K/AKT‐Mediated Oxidative Stress, Inflammation, and Apoptosis,” International Immunopharmacology 124 (2023): 110876, 10.1016/j.intimp.2023.110876. 37683399

[30] L. Fang , Y. Zhang , Q. Wang , et al., “A Polysaccharide From Huaier Ameliorates Cisplatin Nephrotoxicity by Decreasing Oxidative Stress and Apoptosis via PI3K/AKT Signaling,” International Journal of Biological Macromolecules 139 (2019): 932–943, 10.1016/j.ijbiomac.2019.07.219. 31377293

[31] Y. Ma , J. Hu , C. Song , et al., “Er‐Xian Decoction Attenuates Ovariectomy‐Induced Osteoporosis by Modulating Fatty Acid Metabolism and IGF1/PI3K/AKT Signaling Pathway,” Journal of Ethnopharmacology 301 (2023): 115835, 10.1016/j.jep.2022.115835. 36252878

[32] L. Xin , H. C. Feng , Q. Zhang , et al., “Exploring the Osteogenic Effects of Simiao Wan Through Activation of the PI3K/AKT Pathway in Osteoblasts,” Journal of Ethnopharmacology 338 (2025): 119023, 10.1016/j.jep.2024.119023. 39489361

[33] N. K. Y. Wee , N. E. McGregor , E. C. Walker , et al., “Direct Activation of PI3K in Osteoblasts and Osteocytes Strengthens Murine Bone Through Sex‐Specific Actions on Cortical Surfaces,” Journal of Bone and Mineral Research: The Official Journal of the American Society for Bone and Mineral Research 39 (2024): 1174–1187, 10.1093/jbmr/zjae102. 38959852

[34] C. Jin , L. Jia , Z. Tang , and Y. Zheng , “Long Non‐Coding RNA MIR22HG Promotes Osteogenic Differentiation of Bone Marrow Mesenchymal Stem Cells via PTEN/AKT Pathway,” Cell Death & Disease 11 (2020): 601, 10.1038/s41419-020-02813-2. 32732881 PMC7393093

[35] J. Li , Z. Zhong , J. Yuan , X. Chen , Z. Huang , and Z. Wu , “Resveratrol Improves Endothelial Dysfunction and Attenuates Atherogenesis in Apolipoprotein E‐Deficient Mice,” Journal of Nutritional Biochemistry 67 (2019): 63–71, 10.1016/j.jnutbio.2019.01.022. 30856465

[36] L. A. Stechschulte , P. J. Czernik , Z. C. Rotter , et al., “PPARG Post‐Translational Modifications Regulate Bone Formation and Bone Resorption,” EBioMedicine 10 (2016): 174–184, 10.1016/j.ebiom.2016.06.040. 27422345 PMC5006645

[37] S. Baroi , P. J. Czernik , M. P. Khan , et al., “PPARG in Osteocytes Controls Cell Bioenergetics and Systemic Energy Metabolism Independently of Sclerostin Levels in Circulation,” Molecular Metabolism 88 (2024): 102000, 10.1016/j.molmet.2024.102000. 39074536 PMC11367276

[38] X. Wang , M. Zhu , J. J. Loor , et al., “Propionate Alleviates Fatty Acid‐Induced Mitochondrial Dysfunction, Oxidative Stress, and Apoptosis by Upregulating PPARG Coactivator 1 Alpha in Hepatocytes,” Journal of Dairy Science 105 (2022): 4581–4592, 10.3168/jds.2021-21198. 35181129

[39] W. Li , X. Yin , Y. Yan , C. Liu , and G. Li , “Kurarinone Attenuates Hydrogen Peroxide‐Induced Oxidative Stress and Apoptosis Through Activating the PI3K/Akt Signaling by Upregulating IGF1 Expression in Human Ovarian Granulosa Cells,” Environmental Toxicology 38 (2023): 28–38, 10.1002/tox.23659. 36114797

[40] P. Backeljauw , P. Bang , P. E. Clayton , M. Geffner , and K. A. Woods , “Diagnosis and Management of Primary Insulin‐Like Growth Factor‐I Deficiency: Current Perspectives and Clinical Update,” Pediatric Endocrinology Reviews: PER 7 Suppl 1, no. Suppl 1 (2010): 154–171. 20463651

[41] R. Yang , J. Li , J. Zhang , et al., “17β‐Estradiol Plays the Anti‐Osteoporosis Role via a Novel ESR1‐Keap1‐Nrf2 Axis‐Mediated Stress Response Activation and Tmem119 Upregulation,” Free Radical Biology and Medicine 195 (2023): 231–244, 10.1016/j.freeradbiomed.2022.12.102. 36592659

[42] L. Pelusi , D. Mandatori , N. Di Pietrantonio , et al., “Estrogen Receptor 1 (ESR1) and the Wnt/β‐Catenin Pathway Mediate the Effect of the Coumarin Derivative Umbelliferon on Bone Mineralization,” Nutrients 14 (2022): 3209, 10.3390/nu14153209. 35956385 PMC9370350

[43] B. Yu , Z. Mai , X. Liu , and Z. Huang , “Selective Estrogen Receptor Modulator BC‐1 Activates Antioxidant Signaling Pathway in Vitro via Formation of Reactive Metabolites,” Acta Pharmacologica Sinica 34 (2013): 373–379, 10.1038/aps.2012.168. 23334240 PMC4002492

[44] S. Li , X. Li , F. He , R. Jiao , S. Zhang , and Z. Li , “Amarogentin Promotes Osteoblast Differentiation in Oestrogen‐Deficiency‐Induced Osteoporosis Rats by Modulating the Nrf‐2/MAPK/ERK Signalling Pathway,” Archives of Medical Science 19 (2023): 452–457, 10.5114/aoms.2019.89652. 37034504 PMC10074178

[45] Y. Xiao , B. Li , and J. Liu , “MicroRNA‑148a Inhibition Protects Against Ovariectomy‑Induced Osteoporosis Through PI3K/AKT Signaling by Estrogen Receptor Α,” Molecular Medicine Reports 17 (2018): 7789–7796, 10.3892/mmr.2018.8845. 29620276

[46] H. Li , T. Li , J. Fan , et al., “miR‐216a Rescues Dexamethasone Suppression of Osteogenesis, Promotes Osteoblast Differentiation and Enhances Bone Formation, by Regulating c‐Cbl‐Mediated PI3K/AKT Pathway,” Cell Death & Differentiation 22 (2015): 1935–1945, 10.1038/cdd.2015.99. 26206089 PMC4816120

[47] N. Song , Z. Zhao , X. Ma , et al., “Naringin Promotes Fracture Healing Through Stimulation of Angiogenesis by Regulating the VEGF/VEGFR‐2 Signaling Pathway in Osteoporotic Rats,” Chemico‐Biological Interactions 261 (2017): 11–17, 10.1016/j.cbi.2016.10.020. 27833010

[48] T. Behl and A. Kotwani , “Exploring the Various Aspects of the Pathological Role of Vascular Endothelial Growth Factor (VEGF) in Diabetic Retinopathy,” Pharmacological Research 99 (2015): 137–148, 10.1016/j.phrs.2015.05.013. 26054568

[49] Y. Liu , A. D. Berendsen , S. Jia , et al., “Intracellular VEGF Regulates the Balance Between Osteoblast and Adipocyte Differentiation,” Journal of Clinical Investigation 122 (2012): 3101–3113, 10.1172/jci61209. 22886301 PMC3428080

[50] J. C. Liu , C. J. Lengner , T. Gaur , et al., “Runx2 Protein Expression Utilizes the Runx2 P1 Promoter to Establish Osteoprogenitor Cell Number for Normal Bone Formation,” Journal of Biological Chemistry 286 (2011): 30057–30070, 10.1074/jbc.M111.241505. 21676869 PMC3191046

